# Relationship of *TRIM5* and *TRIM22* polymorphisms with liver disease and HCV clearance after antiviral therapy in HIV/HCV coinfected patients

**DOI:** 10.1186/s12967-016-1005-7

**Published:** 2016-09-02

**Authors:** Luz M. Medrano, Norma Rallón, Juan Berenguer, María A. Jiménez-Sousa, Vicente Soriano, Teresa Aldámiz-Echevarria, Amanda Fernández-Rodríguez, Marcial García, Francisco Tejerina, Isidoro Martínez, José M. Benito, Salvador Resino

**Affiliations:** 1 Unidad de Infección Viral e Inmunidad, Centro Nacional de Microbiología, Instituto de Salud Carlos III (Campus Majadahonda), Carretera Majadahonda- Pozuelo, Km 2.2, 28220 Majadahonda Madrid, Spain; 2 Instituto de Investigación Sanitaria de La Fundación Jiménez Díaz (IIS-FJD), Universidad Autónoma de Madrid, Madrid, Spain; 3 Hospital Universitario Rey Juan Carlos, Móstoles, Spain; 4 Unidad de Enfermedades Infecciosas/VIH, Hospital General Universitario “Gregorio Marañón”, Madrid, Spain; 5 Instituto de Investigación Sanitaria Gregorio Marañón (IiSGM), Madrid, Spain; 6 Unidad de Enfermedades Infecciosas, Hospital Universitario La Paz, Madrid, Spain

**Keywords:** TRIM22, TRIM5, SNPs, AIDS, Fibrosis, HCV therapy

## Abstract

**Background and aims:**

TRIM5 and TRIM22 are restriction factors involved in innate immune response and exhibit anti-viral activity. Single nucleotide polymorphisms (SNPs) at *TRIM5* and *TRIM22* genes have shown to influence several viral infections such as human immunodeficiency virus (HIV), hepatitis B, as well as measles and rubella vaccination. The aim of this study is to analyze whether *TRIM5* and *TRIM22* polymorphisms are associated with liver fibrosis inflammation-related biomarkers and response to pegylated-interferon-alpha plus ribavirin (pegIFNα/RBV) therapy in HIV/hepatitis C virus (HCV) coinfected patients.

**Methods:**

A retrospective study was performed in 319 patients who started pegIFNα/RBV therapy. Liver fibrosis stage was characterized in 288 patients. *TRIM5* rs3824949 and *TRIM22* polymorphisms (rs1063303, rs7935564, and rs7113258) were genotyped using the GoldenGate assay. The primary outcomes were: a) significant liver fibrosis (≥F2) evaluated by liver biopsy or transient elastography (liver stiffness values ≥7.1 Kpa); b) sustained virological response (SVR) defined as no detectable HCV viral load (<10 IU/mL) at week 24 after the end of the treatment. The secondary outcome variable was plasma chemokine levels.

**Results:**

Patients with *TRIM5* rs3824949 GG genotype had higher SVR rate than patients with *TRIM5* rs3824949 CC/CG genotypes (p = 0.013), and they had increased odds of achieving SVR (adjusted odds ratio (aOR = 2.58; p = 0.012). Patients with *TRIM22* rs1063303 GG genotype had higher proportion of significant liver fibrosis than patients with rs1063303 CC/CG genotypes (p = 0.021), and they had increased odds of having significant hepatic fibrosis (aOR = 2.19; p = 0.034). Patients with *TRIM22* rs7113258 AT/AA genotype had higher SVR rate than patients with rs7113258 TT genotypes (p = 0.013), and they had increased odds of achieving SVR (aOR = 1.88; p = 0.041). The *TRIM22* haplotype conformed by rs1063303_C and rs7113258_A was more frequent in patients with SVR (p = 0.018) and was significantly associated with achieving SVR (aOR = 2.80; p = 0.013). The *TRIM5* rs3824949 GG genotype was significantly associated with higher levels of GRO-α (adjusted arithmetic mean ratio ((aAMR) = 1.40; p = 0.011) and MCP-1 (aAMR = 1.61; p = 0.003).

**Conclusions:**

*TRIM5* and *TRIM22* SNPs are associated to increased odds of significant liver fibrosis and SVR after pegIFNα/RBV therapy in HIV/HCV coinfected patients. Besides, *TRIM5* SNP was associated to higher baseline levels of circulating biomarkers GRO and MCP-1.

**Electronic supplementary material:**

The online version of this article (doi:10.1186/s12967-016-1005-7) contains supplementary material, which is available to authorized users.

## Background

The natural history of chronic hepatitis C (CHC) is highly variable in progression rates after decades with hepatitis C virus (HCV) infection. Disease progression may vary from minimal changes to advanced fibrosis, cirrhosis, end-stage liver disease, hepatocellular carcinoma, and liver related death [1]. The development of hepatic fibrosis in CHC is multi-factorial and many co-factors, which increase the individual risk of progression, have been identified [2]. In this regard, human immunodeficiency virus (HIV) is the most important co-infection factor identified [2]. Hepatitis C and HIV share routes of transmission, and HIV/HCV co-infection is quite common [1]. Co-infection with HIV and HCV has a negative impact on the natural history of HCV because HIV accelerates the risk of liver disease progression [3, 4], roughly 34 % of co-infected patients increase at least one METAVIR fibrosis stage over 2.5 years [5].

Staging of liver fibrosis is essential for adequate management of patients with CHC, because it provides prognostic information and facilitates decisions on therapy [6, 7]. To date, hepatic biopsy is the gold standard to diagnosis and quantification of liver fibrosis [8]. In order to assess liver biopsy specimens, several systems have been developed, being METAVIR scoring system one of the most widely used [9], which ranks fibrosis on a 5-point scale from F0 (no fibrosis) to F4 (cirrhosis).

The standard of care for CHC was pegylated-interferon-alpha plus ribavirin (pegIFNα/RBV) during many years [10], but this treatment limited the rate of sustained virological response (SVR) and severe associated adverse events, particularly in HIV/HCV coinfected patients [11]. Today, the new IFN-free therapies with direct-acting antivirals (DAAs) display SVR rates above 95 %, including HIV/HCV co-infected patients [12]. Exceptionally, pegIFNα/RBV or pegIFNα/RBV plus DAAs may be used in groups of patients [13]. Therefore, the use of pegIFNα/RBV has dropped drastically and it is no longer recommended therapy for HIV/HCV coinfected patients. However, the access to the newer HCV drugs may be limited in certain countries or regions due to its high cost.

The tripartite motif (TRIM) family, RING finger E3 ubiquitin ligases, comprise around 100 host restriction factors with a potent antiviral activity against HIV [14] and other viruses such as HCV [15]. The restriction factors are part of the innate immune response and normally respond to IFN stimulation [IFN-stimulated genes (ISGs)] [16, 17]. TRIM proteins are also involved in apoptosis, transcription, differentiation and regulation of cell cycle progression, and some TRIM proteins exhibit anti-viral activity [18, 19]. TRIM5 is well known to restrict the HIV-1 infection at an early-stage of reverse transcription [14]. Furthermore, TRIM5 is an E3 ubiquitin ligase that promotes activation of nuclear factor kappa-light-chain-enhancer of activated B cells (NF-κB) and activator protein 1 (AP-1), which play an important role in innate immune response [20, 21]. TRIM22 protein, as well as TRIM5, is an ISG upregulated upon IFN administration to HCV-infected patients and it is also able to induce innate signaling pathways [15, 22]. TRIM22 is a natural antiviral effector of HIV-1 [23]. The TRIM22 expression has been negatively correlated with low HIV-1 plasma viral load and high levels of CD4+ T cell count [24]. Moreover, there is also evidence that TRIM22 is involved in blocking replication of HCV [15, 25] and hepatitis B virus (HBV) [26].

Genetic factors may play an important role for HCV treatment response and disease progression in CHC [27], such as polymorphisms located around *IL28B* region that have been described as predictors of spontaneous HCV clearance and CHC treatment [28]. *TRIM5* and *TRIM22* genes are adjacent and located on chromosome 11 [29]. Single nucleotide polymorphisms (SNPs) at *TRIM5* and *TRIM22* genes have been implicated in several infections such as HIV [14, 30], HBV [31], and measles and rubella vaccination [32, 33]. However, there are not any data about the influence of *TRIM5* and *TRIM22* SNPs on CHC.

The aim of this study was to analyze whether *TRIM5* and *TRIM22* polymorphisms are associated with liver fibrosis inflammation-related biomarkers and response to pegIFNα/RBV therapy in HIV/HCV coinfected patients.

## Methods

### Patients and study design

We carried out a retrospective study in HIV/HCV coinfected patients who started HCV treatment with pegIFNα/RBV on a regular follow-up at two reference HIV hospitals located in Madrid, Spain: Hospital General Universitario “Gregorio Marañón” and Hospital Carlos III. The study was approved by the Research Ethic Committee of the Instituto de Salud Carlos III and was conducted in accordance with the Declaration of Helsinki. All patients gave their written informed consent.

**Fig. 1:**
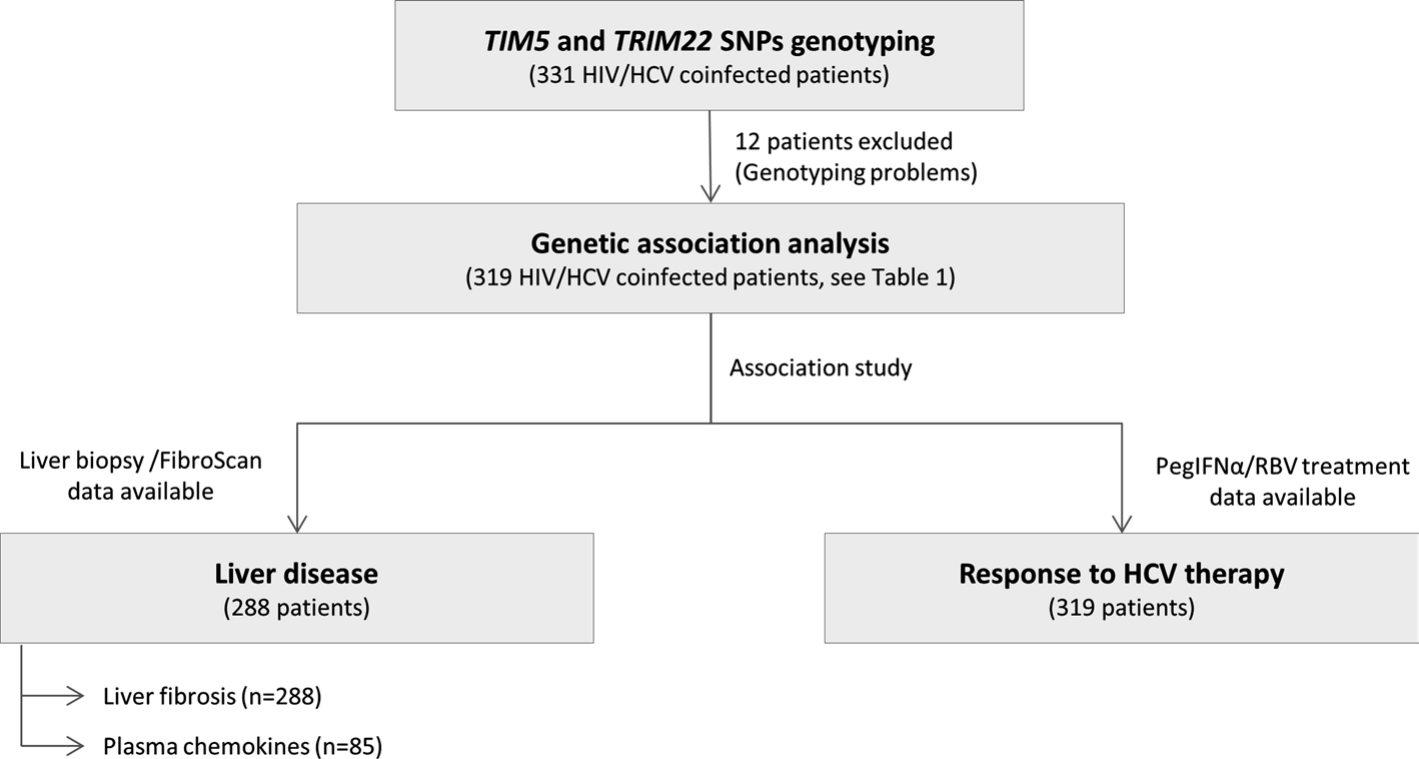
Flow diagram of selection of patients and study design

The inclusion criteria for starting HCV antiviral treatment were: HIV infection, chronic hepatitis C (presence of detectable HCV replication for at least 6 months after HCV infection), negative hepatitis B surface antigen, no clinical evidence of hepatic decompensation, detectable HCV RNA by polymerase chain reaction at baseline, CD4+ count higher than 200 cells/mm^3^, and stable combination antiretroviral therapy (cART) for at least 6 months before study entry or no need according to treatment guidelines used in the study period [10, 34]. The exclusion criteria were: active opportunistic infections, active drug or alcohol addiction, and other concomitant diseases or conditions. A total of 331 patients had available DNA samples, but 12 patients were excluded due to genotyping problems. Finally, 319 patients were available for genetic association analysis (Fig. 1).

### Epidemiological and clinical data

Clinical and epidemiological data were obtained from medical records. Body mass index (BMI) was calculated as the weight in kilograms divided by the square of the height in meters. The duration of HCV infection for patients with a history of intravenous drug use (IDU) was estimated starting from the first year they shared needles and other injection paraphernalia, which are the most relevant risk practices for HCV transmission. For non-IDU patients, we only included those patients for whom the initiation of their HCV infection could be determined with certainty. In this case, the initiation of HCV infection was determined only when patients reported an approximate date of transfusion prior to the introduction of HCV screening on blood donations, surgical intervention, or other HCV risk practices (sexual contact, needle piercing, etc.).

CD4+ cell counts is used to assess the HIV disease stage [35]. HIV-infected patients with a good immune system have a CD4+ count ranges from 500 to 1200 cells/mm^3^. A CD4+ cell counts between 200 and 500 cells/mm^3^ increases the risk for certain infections, such as shingles, thrush, skin infections, bacterial sinus and lung infections, and tuberculosis. A CD4+ count of fewer than 200 cells/mm^3^ is a clinical criterion for diagnosis of stage 3 infection (AIDS).

### Liver biopsy

Liver fibrosis was assessed by different methods, depending on the Hospital: (a) At Hospital General Universitario “Gregorio Marañón” liver biopsy was used and fibrosis was estimated according to Metavir score as follows: F0, non-fibrosis; F1, mild fibrosis; F2, significant fibrosis; F3, advanced fibrosis; and F4, definite cirrhosis. (b) At Hospital Carlos III transient elastography (FibroScan^®^, Echosens, Paris, France) was used, and liver stiffness values ≤7.0, between 7.1 and 9.4, between 9.5 and 12.4, and ≥12.5 were considered to correspond with Metavir scores F0-F1, F2, F3, and F4, respectively [36].

### Hepatitis C therapy

Following both international and national guidelines [10, 34], HCV treatment regimens included pegIFNα 2a or 2b at standard doses (180 µg/week or 1.5 µg/kg/week, respectively) plus weight-adjusted ribavirin dosing (1000 mg/day for patients weighing <75 kg and 1200 mg/day for patients weighing ≥75 kg). The virologic response to HCV treatment was measured by assessing plasma HCV-RNA at 4, 12 weeks, end-of-treatment, and 24 weeks after HCV treatment cessation. Patients with HCV genotypes 1 or 4 received either 48 or 72 weeks of treatment, and patients with HCV genotype 2 or 3 were treated for 24 or 48 weeks, depending on the virologic response at week 4.

### Multiplex ELISA

A multiplex kit (Panomics Afymetrix, Inc.; Procarta^®^ Protein Profiling Assays, Fremont, California, United States) was used to specifically evaluate six plasma biomarkers according to the manufacturer’s specifications using the Luminex 100™ analyser (Luminex Corporation, Austin, TX, United States): IFN-γ-inducible protein 10 (IP-10 or CXCL10), growth-regulated alpha protein (GRO-α or CXCL1), epithelial-derived neutrophil-activating peptide 78 (ENA-78 or CXCL5), eotaxin (CCL11), monocyte chemoattractant protein-1 (MCP-1 or CCL2), and monocyte chemoattractant protein-3 (MCP-3 or CCL7).

### DNA genotyping

Genomic DNA was extracted from peripheral blood with Qiagen kit (QIAamp DNA Blood Midi/Maxi; Qiagen, Hilden, Germany). DNA samples were quantified and sent to the Spanish National Genotyping Center (http://www.cegen.org/) for DNA genotyping by using GoldenGate^®^ assay with VeraCode^®^ Technology (Illumina Inc. San Diego, CA, USA) according to the published Illumina protocol (http://support.illumina.com/content/dam/illumina-support/documents/myillumina/0569bf7e-c9ec-4961-8478-0b103e71eb04/veracode_assay_guide_11312819_revb.pdf). The quality control was performed according to the CeGen criteria, which includes duplicated samples on each plate to check for technical replicates; negative and positive controls in each batch to exclude DNA contamination and ensure a technically correct laboratory process, respectively; genotyping call-rate success over 95 % for all the SNPs and family-based studies to measure the genotyping error rate.

The criteria for selecting *TRIM5* and *TRIM22* polymorphisms were: (i) SNPs located in putative regulatory region; (ii) minor allelic frequency (MAF) greater than 20 % for CEU (Utah residents with ancestry from Northern and Western Europe) and TSI (Toscan in Italy) Hapmap population; (iii) selection of tagSNPs according to linkage disequilibrium (LD) >0.8. Finally, four SNPs were selected: rs3824949 in *TRIM5* and rs7935564, rs1063303 and rs7113258 in *TRIM22*.

### Outcome variables

The outcome variables were: (a) significant liver fibrosis evaluated by liver biopsy (F ≥ 2) or transient elastography (liver stiffness values ≥7.1 Kpa); (b) levels of plasma biomarkers of inflammation; (c) SVR defined as no detectable HCV viral load (<10 IU/mL) at week 24 after the end of the treatment.

### Statistical analysis

For the description of the study population, p values were estimated with nonparametric tests: Mann–Whitney U test was used for continuous variables and Chi squared or Fisher’s exact test (when expected values were below 5) for categorical variables.

The genetic association study was carried out according to the genetic model that best fit our data (additive, recessive or dominant). Logistic regression analysis was used to investigate the relationship of *TRIM5* and *TRIM22* polymorphisms with outcome variables. Each logistic regression test was adjusted by the most significant co-variables associated with each one of the outcome variables, avoiding the over-fitting of the regression. We included the SNP [Enter algorithm (forced entry for the SNP)] and the most relevant characteristics by stepwise algorithm (at each step, factors are considered for removal or entry: a p value for entry and exit of 0.15 and 0.20, respectively). The covariables used were age, gender, BMI, baseline HCV-RNA viral load (<500,000 vs. ≥500,000 IU/mL), significant fibrosis (F < 2 vs. F ≥ 2), *Interleukin*-*28B* (*IL28B)* rs12980275 polymorphism (AA vs. AG/GG), and HCV genotype [GT1/4 vs. GT2/3)]. Moreover, the association between *TRIM5* and *TRIM22* polymorphisms and plasma biomarkers was investigated by using General Lineal Model (log-link) adjusted for the same variables described above. All statistical analyses were performed by using the IBM SPSS Statistics for Windows, Version 22.0 (IBM Corp, Chicago, Armonk, NY, USA).

In addition, pair-wise linkage disequilibrium (LD) analysis was computed by Haploview 4.2 software. Haplotype frequencies were inferred with the Expectation–Maximization algorithm and haplotype-based association testing was performed using PLINK software. All p values were two-tailed and statistical significance was defined as p < 0.05.

### In silico analysis

The in silico analysis for possible functional implications of each polymorphism was evaluated by using four web-tools: (a) VarioWatch (http://genepipe.ncgm.sinica.edu.tw/variowatch/); (b) rSNABase (http://rsnp.psych.ac.cn/); (c) SIFT (http://sift.bii.a-star.edu.sg/index.html); (d) analysis for possible miRNA binding site according to each polymorphism was explored by using MicroSNiPer web tool (http://epicenter.ie-freiburg.mpg.de/services/microsniper/).

## Results

### Characteristics of patients and *TRIM5* and *TRIM22* polymorphisms

The baseline characteristics of 319 HIV/HCV coinfected patients used in the current study are presented in Table 1. The median age was 42 years, 77.1 % were males and 84.0 % were on cART. The median baseline CD4+ count was 461 cells/mm^3^, 76.1 % had plasma HIV-RNA <50 copies/mL, 63.3 % had significant liver fibrosis (≥F2), 26.2 % had plasma HCV-RNA <500,000 UI/mL, and 45.8 % had the favorable genotype of *IL28B* polymorphism (AA rs12980275).

**Table 1:** Main epidemiological and clinical characteristics of HIV/HCV coinfected patients on HCV antiviral therapy

Characteristics	All patients
No.	319
Male	246 (77.1 %)
Age (years)	42 (38.7–45.9)
Anthropometric values	
Height (m)	1.7 (1.6–1.7)
Weight (kg)	67 (60–75)
BMI (kg/m^2^)	23.1 (21.2–25.4)
IVDU	281 (89.8 %)
Time of HCV infection (months)	19.6 (12.7–23.5)
cART	268 (84.0 %)
HIV markers	
Nadir CD4+ T-cells/μL	226 (132–341)
Nadir CD4+ <200 cells/μL	135 (42.3 %)
Baseline CD4+ T-cells/μL (n = 316)	461 (364–670)
Baseline CD4+ <500 T-cells/μL (n = 316)	175 (55.4 %)
HIV-RNA <50 copies/ml (n = 314)	239 (76.1 %)
HCV markers	
HCV genotypes (n = 317)	
GT 1	179 (56.5 %)
GT 2	1 (0.3 %)
GT 3	100 (31.5 %)
GT 4	37 (11.7 %)
HCV-RNA (n = 313)	
HCV-RNA <500,000 IU/mL	82 (26.2 %)
Log_10_ HCV-RNA (IU/mL)	6.1 (5.6–6.8)
*IL28B* polymorphism (rs12980275)	
AA	146 (45.8 %)
AG	146 (45.8 %)
GG	27 (8.5 %)
Liver fibrosis (n = 288)	
Significant fibrosis (F ≥ 2)	182 (63.3 %)
Advanced fibrosis (F ≥ 3)	98 (33.9 %)

Values expressed as absolute number (percentage) and median (interquartile range)

*BMI* body mass index; *IVDU* intravenous drug users; *HCV* hepatitis C virus; *HCV-RNA* HCV serum viral load; *GT* HCV genotype; *HIV-1* human immunodeficiency virus type 1; *HIV-RNA* HIV plasma viral load; *cART* combination antiretroviral therapy

The allelic and genotypic frequencies for *TRIM5* and *TRIM22* polymorphisms in HIV/HCV coinfected patients are shown in Additional file 1: Table S1. The MAF was >5 % and genotyping call-rate success was over 95 % for all the SNPs. The frequencies in our dataset were in accordance with the data listed on the NCBI SNP database (http://www.ncbi.nlm.nih.gov/projects/SNP/). Furthermore, *TRIM5* and *TRIM22* polymorphisms were in Hardy–Weinberg equilibrium (p > 0.05), except for *TRIM22* rs7935564 (p = 0.0035) which was discarded for the analysis of genetic association.

### Genetic association of *TRIM5* and *TRIM22* polymorphisms

The association of *TRIM5* and *TRIM22* polymorphisms with outcome variables [significant fibrosis and SVR] in HIV/HCV coinfected patients are shown in Table 2. Patients with *TRIM5* rs3824949 GG genotype had higher SVR rate than patients with *TRIM5* rs3824949 CC/CG genotypes (p = 0.013), and they had increased odds of achieving SVR (adjusted odds ratio (aOR = 2.58; p = 0.012). Patients with *TRIM22* rs1063303 GG genotype had higher proportion of significant fibrosis than patients with rs1063303 CC/CG genotypes (p = 0.021), and they had increased odds of having significant fibrosis (aOR = 2.19; p = 0.034). Additionally, when we stratified our data by baseline HCV-RNA viral load (<500,000 vs. ≥500,000 IU/mL), we observed an almost significant association between rs1063303 GG genotype and significant fibrosis in patients with high HCV viral load (aOR = 2.18; p = 0.070). Moreover, patients with *TRIM22* rs7113258 AT/AA genotype had higher SVR rate than patients with rs7113258 TT genotypes (p = 0.013), and they had increased odds of achieving SVR (aOR = 1.88; p = 0.041).

**Table 2:** Association of *TRIM5* rs3824949 and *TRIM22* rs1063303, rs7113258 polymorphism with significant fibrosis (F ≥ 2) at baseline and sustained virological response (SVR) in HIV/HCV coinfected patients on HCV therapy

	Unadjusted			Adjusted	
	CC/CG	GG	p value^a^	**a**OR (95 % CI)	p value^b^
***TRIM5*** ** rs3824949 (Recessive)**					
F ≥ 2 (n = 287)	31.6 % (142/225)	48.7 % (39/62)	0.976	0.84 (0.46–1.55)	0.589
SVR (n = 318)	50.8 % (127/250)	67.6 % (46/68)	*0.013*	2.58 (1.23–5.39)	*0.012*
***TRIM22*** ** rs1063303 (Recessive)**					
F ≥ 2 (n = 286)	59.8 %(140/234)	76.9 % (40/52)	*0.021*	2.19 (1.06–4.53)	*0.034*
SVR (n = 317)	55.6 % (145/261)	48.2 % (27/56)	0.317	0.89 (0.41–1.91)	0.760

*TRIM22* rs7113258 (Dominant)	TT	AT/AA	p value^a^	**a**OR (95 % CI)	p value^b^
F ≥ 2 (n = 287)	63.3 % (124/196)	63.7 % (58/91)	0.939	1.06 (0.62; 1.82)	0.829
SVR (n = 317)	49.8 % (107/215)	64.7 % (66/102)	*0.013*	1.88 (1.03–3.38)	*0.041*

Categorical variables are expressed in percentage (absolute count)

Statistically significant differences are shown in italics

*aOR* adjusted odds ratio; *95* *%CI* 95 % confidence interval; *HCV* hepatitis C virus; *HIV* human immunodeficiency virus. *TRIM5* tripartite motif-containing 5; *TRIM22* tripartite motif-containing 22

^a^p values were calculated by Chi square tests or Fisher’s exact test when expected values are below five

^b^p cvalues were calculated by logistic regression adjusting for the most important clinical and epidemiological characteristics (see statistical analysis section)

Haplotype frequencies of *TRIM22* polymorphism (rs1063303 and rs7116258) stratified by outcome variables are presented in Table 3. The allelic combination conformed by rs1063303_C and rs7113258_A was more frequent in patients with SVR (p = 0.018) and was significantly associated with the achievement of SVR (aOR = 2.80; p = 0.013).

**Table 3:** Haplotype frequencies of *TRIM22* rs1063303 and rs7113258 polymorphisms and their genetic association with significant fibrosis (F ≥ 2) at baseline and sustained virological response (SVR) in HIV/HCV coinfected patients on HCV therapy

Haplotypes by fibrosis	Frequency		OR (95 % CI)	p value^a^	**a**OR (95 % CI)	p value^b^
	F < 2 (%)	F ≥ 2 (%)				
CT	49.5	47.0	0.88 (0.61–1.28)	0.509	0.91 (0.62–1.34)	0.627
GT	34.8	35.7	1.05 (0.71–1.55)	0.818	1.03 (0.68–1.54)	0.902
GA	8.6	8.2	0.94 (0.46–1.95)	0.876	0.88 (0.41–1.89)	0.749
CA	7.1	9.1	1.43 (0.69–2.95)	0.321	1.52 (0.68–3.37)	0.294

Haplotypes by HCV treatment response	Non-SVR (%)	SVR (%)	OR (95 % CI)	p value^a^	**a**OR (95 % CI)	p value^b^
CT	50.7	46.5	0.82 (0.58–1.15)	0.244	0.78 (0.51–1.18)	0.236
GT	36.5	33.3	0.84 (0.58–1.21)	0.345	0.86 (0.55–1.34)	0.496
GA	7.0	10.0	1.81 (0.90–3.61)	0.090	1.61 (0.67–3.88)	0.286
CA	5.9	10.2	2.29 (1.12–4.70)	*0.018*	2.80 (1.19–6.57)	*0.013*

Statistically significant differences are shown in italics

*aOR* adjusted odds ratio; *95* *%CI* 95 % confidence interval; *SVR* sustained virological response; *HCV* hepatitis C virus; *HIV* human immunodeficiency virus

^a^p values were calculated by Chi square tests

^b^p values were calculated by logistic regression adjusting for the most important clinical and epidemiological characteristics (see ‘‘statistical analysis’’ section)

### Plasma chemokines

The association of *TRIM5* rs3824949 polymorphism with levels of plasma biomarkers in HIV/HCV coinfected patients are shown in Table 4. Patients with rs3824949 GG genotype had higher plasma levels of eotaxin (p = 0.038) and MCP-1 (p = 0.034) than patients carrying rs3824949 CC/CG genotypes. Besides, patients with rs3824949 GG genotype tended to have higher levels of GRO-α (p = 0.064). When multivariate analysis was performed, rs3824949 GG genotype was significantly associated with higher levels of GRO-α [adjusted arithmetic mean ratio (aAMR) = 1.40; p = 0.011] and MCP-1 (aAMR = 1.61; p = 0.003).

**Table 4:** Association of *TRIM5* rs3824949 polymorphism with levels of plasma biomarkers in HIV/HCV coinfected patients at baseline

	CC/CG	GG	p value^a^	**a**AMR (95 % CI)	p value^b^
IP-10 or CXCL10 (pg/ml)	517.7 (573.8)	696.5 (979.4)	0.510	1.56 (0.94; 2.59)	0.082
GRO-α or CXCL1 (pg/ml)	62.6 (47.3)	80.4 (33.0)	0.064	1.40 (1.08; 1.83)	*0.011*
ENA-78 o CXCL5 (pg/ml)	93.4 (103.9)	114.2 (140.4)	0.391	1.26 (0.90; 1.76)	0.165
Eotaxin or CCL11 (pg/ml)	43.8 (55.5)	78.3 (67.5)	*0.038*	1.19 (0.89; 1.61)	0.234
MCP-1 or CCL2 (pg/ml)	28.5 (42.1)	64.3 (82.9)	*0.034*	1.61 (1.18; 2.19)	*0.003*
MCP-3 or CCL7 (pg/ml)	16.2 (17.6)	18.6 (15.7)	0.973	0.91 (0.66; 1.24)	0.543

Data are expressed in median (interquartile range)

Statistically significant differences are shown in italics

*aAMR* adjusted arithmetic mean ratio; *95* *%CI* 95 % confidence interval; *IP-10 or CXCL10* IFN-γ-inducible protein 10; *GRO-α or CXCL1* growth-regulated alpha protein; *ENA-78 or CXCL5* epithelial-derived neutrophil-activating peptide 78; *CCL11* eotaxin; *MCP-1 CCL2* monocyte chemoattractant protein; *CCL7* MCP-3; *HCV* hepatitis C virus; *HIV* human immunodeficiency virus

^a^p values were calculated by Mann–Whitney test

^b^p values were calculated by General Lineal Model (GLM) after adjusting by the most important clinical and epidemiological characteristics (see ‘‘statistical analysis’’ section)

We did not find any significant differences in plasma biomarker levels according to the genotypes of *TRIM22* rs1063303 and rs7113258 (data not shown).

## Discussion

The genetic variation of genes involved in innate immune system may influence the odds of clinical outcomes in CHC and HIV infection [27, 37]. In our study, patients with *TRIM22* rs1063303 polymorphism was related to higher odds of having significant fibrosis, whereas *TRIM5* rs3824949 and *TRIM22* rs7113258 polymorphisms were associated with achieving SVR and higher plasma levels of inflammation biomarkers.

Immunological response has a crucial role in viral persistence and liver damage during CHC [38, 39]. The development of fibrosis and cirrhosis depends on the functional diversity of this response and the balance between pro-inflammatory and anti-inflammatory response [39]. TRIM proteins are encoded by ISGs that are involved in the regulation of anti-viral innate immune response [18, 19]. TRIM proteins have a key role in the regulation of pathways downstream of viral RNA and DNA sensors, and in the inflammasome; besides, TRIMs may contribute to the development and pathology of autoimmune and autoinflammatory conditions [40].

TRIM22 is an interferon-induced protein that potently inhibits the replication of diverse viruses such as HIV-1 [23], HCV [15, 25] and HBV [26]; and the overexpression of TRIM22 activates NF-κB in a dose-dependent manner [41], promoting autoimmune diseases [42]. *TRIM22* SNPs have been related to several aspects of viral infections such as HIV replication [30], chronic hepatitis B infection [31], and levels of specific antibodies and cytokines following measles and rubella vaccination [32, 33]. The *TRIM22* rs1063303 G > C causes an arginine (R) to threonine (T) amino acid change at position 242 in the TRIM22 protein [43]. The *TRIM22* rs1063303 GG variant correlated to an inverse functional impact where it increased TRIM22 expression and decreased the antiviral activity of TRIM22 [43]. In this regard, the overexpression of TRIM22 negatively correlated with HCV viral load [25] and HIV-1 viral load [44], while gene silencing of TRIM22 enhanced HIV-1 infection of target cells [44]. Besides, the rs1063303 GG genotype has also been associated with a more efficient HIV-1 replication [30]. In our study, the rs1063303 GG genotype was associated with significant fibrosis. Furthermore, we found an almost significant association between rs1063303 GG genotype and significant fibrosis only in patients with high HCV viral load. It is possible that rs1063303 GG genotype might impair the control of HCV replication and could favor a more powerful inflammatory response, which could promote more efficiently the development of liver fibrosis.

Moreover, patients with *TRIM22* rs7113258 TA/AA genotype had increased odds of achieving SVR after pegIFNα/RBV therapy. The rs7113258 polymorphism is located at a regulatory region [3′ untranslated region (UTR)]. Therefore, it could have a regulatory effect on the *TRIM22* gene expression. In this setting, we analyzed in silico whether this *TRIM22* rs7113258 could be part of microRNAs (miRNAs) binding sites via MicroSNiPer [45]. The miRNAs are negative gene regulators influencing gene expression by binding at the 3′UTR level [46]. We found that rs7113258 A allele generates putative target sites for several miRNAs (hsa-miR-4495, hsa-miR-3668 and hsa-miR-3148), whereas the presence of rs7113258 T allele disrupts these target sites and generates others (hsa-miR-4678 and hsa-miR-3177-5p). Thus, we might hypothesize that these differences in the miRNAs binding between rs7113258 genotypes could be implicated in the observed association. However, further studies investigating the functional role of this SNP would be interesting and it should not be excluded that rs7113258 could be just a tagSNP in LD with the causal variant.

The LD analysis showed that there was low LD (non-random association of alleles at different loci) between *TRIM22* SNPs (D’ = 0.14), meaning that there is evidence of a possible recombination between these SNPs. Moreover, the R-squared among *TRIM22* SNPs was low (R^2^ = 0.01), meaning that the *TRIM22* SNPs did not provide exactly the same information and could not be substituted one for the other. Taking into account these results, it is very important to perform allelic combination analysis between these two variants in order to better explore the genetic variance in this region. We observed that the allelic combination conformed by rs1063303_C and rs7113258_A increased the likelihood of SVR after pegIFNα/RBV therapy, with a higher odd ratio (OR = 2.80) than rs7113258 alone (OR = 1.88). Though we did not observe any association between *TRIM22* rs1063303 CC genotype and SVR, which may be due to the limited sample size. Thus, our study could have enough statistic power to detect differences in rs1063303 when combined with rs7113258 because more genetic variability is captured, but not when this SNP is analyzed alone. The allelic combinations in *TRIM22* give us an increased knowledge about possible genetic effect in the loci. The causal variant in the region could be in LD with the combination of rs1063303_C-rs7113258_A.

On the other hand, TRIM5 protein restricts the HIV-1 replication but their exact antiviral mechanism remains unclear [14]. Furthermore, TRIM5 could act as a pattern recognition receptor that triggers the E3-ubiquitin ligase activity, leading to the activation of the NF-κB and AP-1 and promoting innate immune response [14]. Other *TRIM5* polymorphisms (rs10838525 and rs3740996) have been related to higher resistance to HIV-1 infection [14] and *TRIM5* rs3824949 GG is associated with higher levels of antibodies in response to measles and rubella vaccines [32, 33]. Our results show that patients with *TRIM5* rs3824949 GG genotype had higher likelihood of achieving SVR. The *TRIM5* rs3824949 polymorphism is located at 5′ UTR. 5′ UTR region is already known to play crucial roles in the post-transcriptional regulation of gene expression [47]. We analyzed in silico whether this *TRIM5* rs3824949 could be a part of RNA binding protein site via rSNPBase [48]. We found that rs3824949 is involved in RNA binding protein-mediated post-transcriptional regulation. Additionally, rs3824949 shows an eQTL (expression quantitative trait loci) effect, meaning that there is evidence for a direct functional relationship between the rs3824949 polymorphism and the measured expression levels of different genes. It is possible that the rs3824949 polymorphism may influence the expression of *TRIM5* gene at the level of translation, favoring HCV eradication after pegIFNα/RBV therapy.

In addition, patients with *TRIM5* rs3824949 GG genotype had higher levels of plasma chemokines. GRO-α (CXCL-1), MCP-1 (CCL-2), and eotaxin (CCL-11) are members of the chemokine family, which are secreted by the liver during chronic HCV infection, and their levels are strongly associated with lymphocyte recruitment, immune response, and fibrogenesis [49, 50]. CXCL-1 is a potent agonist for CXCR2 which recruits neutrophils to the site of inflammation or injury during liver disease [51], and plasma levels of CXCL1 are associated with liver fibrosis in HCV-infected patients [52]. MCP-1 is a potent agonist for CCR2, this chemokine participates in regulating Th2 lymphocyte subset infiltration in liver [49, 50]. CCL-11 is a chemokine that selectively recruits eosinophils, basophils and Th2 lymphocytes through CCR3 [53]. Then, patients with rs3824949 GG genotype seem to have a predominant Th2 immune profile. Moreover, it has been reported that non-responder patients to IFN-α therapy show preactivation of their IFN system and have some defects at steps downstream of ISG expression, making them refractory to both endogenous IFN-α and IFN-α therapy [54]. Thus, these chemokines may reflect the degree of Th2 immune responses, and under this scenario, patients with an increased Th2 profile may indicate a healthy Th1 immune response and low preactivation of their IFN pathway, making them more responsive to pegIFNα/RBV therapy.

Finally, several aspects have to be taken into account for the correct interpretation of the results. Firstly, this report has a retrospective design with a relatively small number of patients, which could limit the statistical power to detect differences between groups. Secondly, the patients selected for our study were patients who met a set of criteria for starting HCV treatment, and it is possible that this may have introduced a selection bias. Thirdly, we had no access to HCV-monoinfected patients in order to evaluate the influence of *TRIM5* and *TRIM22* polymorphisms on these patients. Moreover, since the study was carried out entirely in European whites, and the frequency of these alleles differs among different ethnicities, it would be necessary to perform an independent replication of this study for different ethnic groups.

## Conclusion

In summary, the major findings were: (1) The *TRIM22* rs1063303 GG genotype was associated with having significant fibrosis; (2) The *TRIM5* rs3824949 GG genotype and *TRIM22* rs7113258 TA/AA genotypes were associated with achieving SVR; (3) The *TRIM22* haplotype conformed by rs1063303_C and rs7113258_A was associated with achieving SVR; (4) The *TRIM5* rs3824949 GG genotype was associated with having high plasma levels of GRO-α and MCP-1. These data suggest that *TRIM2* and *TRIM5* polymorphisms may play a major role in pathogenesis of CHC in HIV/HCV coinfected patients. Further researches are necessary to corroborate these findings.

## Additional file

10.1186/s12967-016-1005-7 Summary of allelic and genotypic frequencies for *TRIM5* and *TRIM22* polymorphisms in HIV/HCV coinfected patients.

## Abbreviations

CHC: chronic hepatitis C
HCV: hepatitis C virus
HIV: human immunodeficiency virus
pegIFNα/RBV: pegylated interferon-alpha plus ribavirin
SVR: sustained virological response
DAAs: direct-acting antivirals
TRIM: tripartite motif
TRIM5: tripartite motif-containing 5
TRIM22: tripartite motif-containing 22
ISGs: IFN-stimulated genes
NF-κB: nuclear factor kappa-light-chain-enhancer of activated B cells
AP-1: activator protein 1
SNPs: single nucleotide polymorphisms
MAF: minor allele frequency
BMI: body mass index
IDU: intravenous drug use
GRO-α: growth-related oncogene- α
IP-10 or CXCL10: IFN-γ-inducible protein 10
GRO-α or CXCL1: growth-regulated alpha protein
ENA-78 or CXCL5: epithelial-derived neutrophil-activating peptide 78
CCL11: eotaxin
MCP-1 or CCL2: monocyte chemoattractant protein-1
MCP-3 or CCL7: monocyte chemoattractant protein-3
LD: linkage disequilibrium
aOR: adjusted odds ratio
aAMR: adjusted arithmetic mean ratio
cART: combination antiretroviral therapy
IL28B: interleukin 28B
UTR: untranslated region
miRNAs: microRNAs
