# Supplementary material for: Relationship of TRIM5 and TRIM22 polymorphisms with liver disease and HCV clearance after antiviral therapy in HIV/HCV coinfected patients
Source: J Transl Med. 2016 Sep 2;14(1):257. doi: 10.1186/s12967-016-1005-7 (PMC5010694; doi:10.1186/s12967-016-1005-7)
Supplement: Supplementary file 1 — 10.1186/s12967-016-1005-7 Summary of allelic and genotypic frequencies for TRIM5 and TRIM22 polymorphisms in HIV/HCV coinfected patients. [file 12967_2016_1005_MOESM1_ESM.docx]

**Supplemental table 1**. Summary of allelic and genotypic frequencies for *TRIM5* and *TRIM22* polymorphisms in HIV/HCV coinfected patients.

|  | **Count** | **Proportion** |  |
| --- | --- | --- | --- |
| ***TRIM5* rs3824949 (n=318)** |  |  |  |
| **Allele frequencies** |  |  |  |
| C | 347 | 55% |  |
| G | 289 | 45% |  |
| **Genotype frequencies** |  |  |  |
| CC | 97 | 31% |  |
| CG | 153 | 48% |  |
| GG | 68 | 21% |  |
| NA | 1 | - |  |
| **Hardy-Weinberg equilibrium** | P-value= 0.650 | |  |
| ***TRIM22* rs7935564 (n=312)** |  |  |  |
| **Allele frequencies** |  |  |  |
| A | 414 | 66% |  |
| G | 210 | 34% |  |
| **Genotype frequencies** |  |  |  |
| AA | 149 | 48% |  |
| AG | 116 | 37% |  |
| GG | 47 | 15% |  |
| NA | 7 | - |  |
| **Hardy-Weinberg equilibrium** | **P-value= 0.0035** | |  |
| ***TRIM22* rs1063303 (n=317)** |  |  |  |
| **Allele frequencies** |  |  |  |
| C | 359 | 57% |  |
| G | 275 | 43% |  |
| **Genotype frequencies** |  |  |  |
| CC | 98 | 31% |  |
| CG | 163 | 51% |  |
| GG | 56 | 18% |  |
| NA | 2 | - |  |
| **Hardy-Weinberg equilibrium** | P-value = 0.430 | |  |
| ***TRIM22* rs7113258 (n=317)** |  |  |  |
| **Allele frequencies** |  |  |  |
| T | 527 | 83% |  |
| A | 107 | 17% |  |
| **Genotype frequencies** |  |  |  |
| AA | 5 | 2% |  |
| TA | 97 | 31% |  |
| TT | 215 | 68% |  |
| NA | 2 | - |  |
| **Hardy-Weinberg equilibrium** | P-value= 0.160 | |  |

Statistically significant differences are shown in bold. (a), P-values were calculated by Chi-squared test.

**Abbreviations:** *TRIM5*, Tripartite motif-containing 5; *TRIM22*, Tripartite motif-containing 22; NA, not available.
